# Supplementary material for: Utility Analyses of AVITI Sequencing Chemistry
Source: bioRxiv. 2024 Jun 27:2024.04.18.590136. Originally published 2024 Apr 22. Preprint. [Version 2] doi: 10.1101/2024.04.18.590136 (PMC11071311; doi:10.1101/2024.04.18.590136)
Supplement: Supplement 1 — Supplemental Figure 1: Phred score trend line of 16 samples sequenced through AVITI or NextSeq 550. Phred score from each of the 16 E. coli genome sequencings were plotted cycle by cycle. The average score for each cycle was shown. Sequencing through NextSeq 550 or AVITI was indicated. Supplemental Figure 2: Nucleotide error distribution in AVITI and Illumina sequencing. Left panel: Substitution error in each of A, T, C, and G in NextSeq 550 and AVITI platforms. Right panel: Insertion/deletion error in each of A, T, C, and G in NextSeq 550 and AVITI platforms. [file media-1.pptx]

## Slide 1
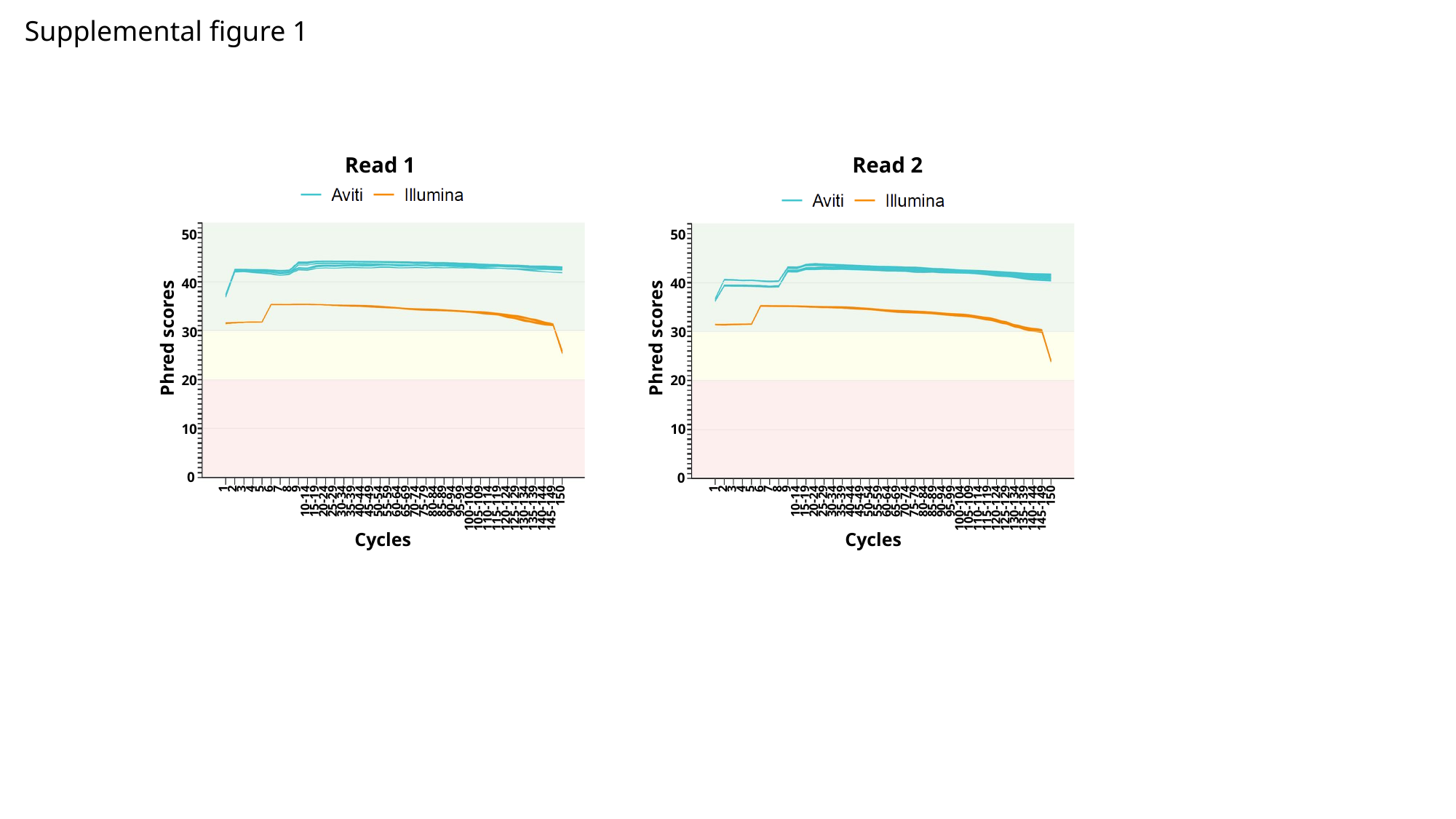

Supplemental figure 1
Read 1			 Read 2
50
50
40
40
30
30
Phred scores
Phred scores
1
2
3
4
5
6
7
8
9
10-14
15-19
20-24
25-29
30-34
35-39
40-44
45-49
50-54
55-59
60-64
65-69
70-74
75-79
80-84
85-89
90-94
95-99
100-104
105-109
110-114
115-119
120-124
125-129
130-134
135-139
140-144
145-149
150
1
2
3
4
5
6
7
8
9
10-14
15-19
20-24
25-29
30-34
35-39
40-44
45-49
50-54
55-59
60-64
65-69
70-74
75-79
80-84
85-89
90-94
95-99
100-104
105-109
110-114
115-119
120-124
125-129
130-134
135-139
140-144
145-149
150
20
20
10
10
0
0
Cycles
Cycles

## Slide 2
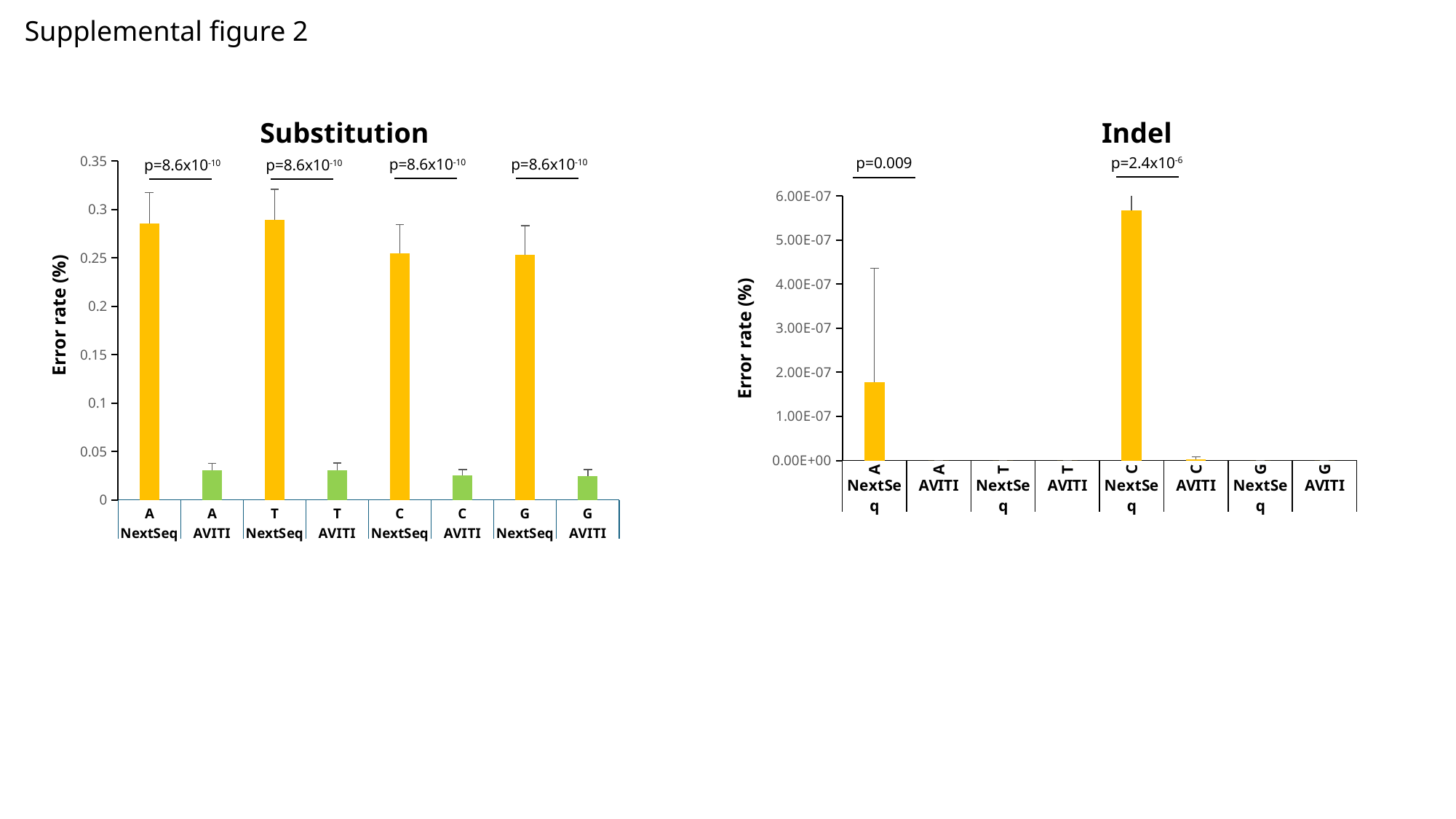

Supplemental figure 2
Substitution					 	 Indel
### Chart
| Category | |
|---|---|
| A | 0.28552 |
| A | 0.030674 |
| T | 0.288951 |
| T | 0.030840000000000003 |
| C | 0.25468399999999997 |
| C | 0.025127 |
| G | 0.252922 |
| G | 0.024783 |p=2.4x10^-6^
p=0.009
p=8.6x10^-10^
p=8.6x10^-10^
p=8.6x10^-10^
p=8.6x10^-10^
### Chart
| Category | |
|---|---|
| A | 1.78e-07 |
| A | 0.0 |
| T | 0.0 |
| T | 0.0 |
| C | 5.68e-07 |
| C | 3.07e-09 |
| G | 0.0 |
| G | 0.0 |Error rate (%)
Error rate (%)
